# Novel therapeutic targets at the crossroad between epigenetics and signal transduction pathways in B-cell lymphomas

**DOI:** 10.1007/s10238-026-02169-5

**Published:** 2026-06-08

**Authors:** Juairia Uddin Dekha, Amir Yami, Laura Quotti Tubi, Iris Haxhiu, Alessandro Carrer, Livio Trentin, Francesco Piazza, Sabrina Manni

**Affiliations:** 1 Department of Medicine-DIMED, University of Padova, Padova, Italy; 2 Veneto Institute of Molecular Medicine, Padova, Italy; 3 Department of Surgery, Oncology and Gastroenterology, University of Padova, Padova, Italy; 4 Department of Biology, University of Padova, Padova, Italy

**Keywords:** Lymphoma, Protein kinase CK1α, Protein kinase CK2, Non-oncogene addiction, Cell signaling, Epigenetics

## Abstract

Lymphomas (Hodgkin lymphoma and non-Hodgkin lymphoma) constitute a heterogenous group of malignancies derived from immature and mature lymphoid cells. Despite improvements in therapeutic options in recent years, high rates of recurrence and resistance to current treatments remain a significant and unmet clinical eliminate s from needs. Past and recent studies have identified important molecular signatures playing a role in the pathophysiology of lymphomas, including the B-cell receptor, NF-κB, PI3K/AKT, Wnt/β-catenin cascades, and have pointed to protein kinases, such as BTK, PI3K, CK1α and CK2, as drivers and therefore potential therapeutic targets. It has also been extensively demonstrated that various epigenetic changes, including DNA methylation and histone modifications, play critical roles in sustaining cell survival, proliferation and perturbed differentiation of lymphoma cells. Moreover, the interplay between signal transduction and epigenetic modifications in lymphoma cells may influence the lymphoma-associated tumor microenvironment and immune cell function, shaping the anti-lymphoma immune response. Therefore, the identification of new targets for the therapy of lymphomas requires a comprehensive understanding of how cell signaling and epigenetic modifications interact. This review explores the function of newly recognized kinases in lymphoma pathogenesis, such as CK1, CK2 and others, highlighting their interaction with epigenetic regulation. The therapeutic potential of targeting simultaneously signaling and epigenetic pathways is proposed as a potential innovative strategy in the treatment of lymphomas.

## Background

Lymphomas are a heterogeneous group of malignant tumors that develop from the aberrant transformation of lymphocytes, which include B, T, and NK cells, at different stages of maturation [1–3]. These malignancies are generally divided into two main groups: Hodgkin lymphoma (HL) and non-Hodgkin lymphoma (NHL**)**, with NHL further subdivided into numerous distinct subtypes, with regard to clinical presentation, treatment approaches, and prognosis [3]. Biologically, lymphoma cells exhibit either slow or rapid-proliferative kinetics, which correlate with their clinical classification as indolent or aggressive subtypes, respectively. Although survival rates have improved markedly over the past decades as a result of advances in diagnostic tools, molecular characterization and treatment, prognosis remains highly variable and depends on lymphoma subtype, disease stage, and patient-specific factors [1, 3–6]. Among mature B cell tumors, NHL are roughly ten times more frequent than HL. In 2025 in USA, estimated new NHL cases are 80,350 (4% of all cancers) and new HL cases are 8720 (American Cancer Society 2025). The most frequent NHL types are Diffuse Large B Cell Lymphoma (DLBCL), Follicular Lymphoma (FL), Marginal Zone Lymphoma (MZL) and Mantle Cell Lymphoma (MCL). The most frequent types of mature T cell lymphomas include Peripheral T-cell lymphomas, not otherwise specified (PTCL, NOS), Anaplastic Large Cell Lymphoma (ALCL) and T-Follicular Helper-derived lymphomas (including angioimmunoblastic lymphoma) [7]. Diagnosis of lymphoma involves clinical evaluation, imaging (such as CT scan, PET), and histopathological assessment of tissue biopsies. Immunophenotyping and molecular diagnostics further refine classification and guide treatment selection [4, 6, 7]. While HL and several indolent NHL are generally associated with favorable outcomes and high survival rates [4, 7, 8], aggressive subtypes often present therapeutic challenges due to rapid progression, relapse or treatment resistance. Even if chemotherapy remains a cornerstone of treatment, particularly in aggressive forms, immunotherapy has significantly advanced the therapeutic landscape, with monoclonal antibodies such as Rituximab and Obinutuzumab (targeting CD20 on B cells), antibody drug conjugates (ADCs), such as Polatuzumab vedotin (targeting CD79b) and Brentuximab vedotin (targeting CD30), immune checkpoint inhibitors (e.g., Nivolumab, Pembrolizumab), bispecific antibodies targeting CD20 and CD3 (Mosunetuzumab, Glofitamab, Epcoritamab, Odronextamab), and chimeric antigen receptor T (CAR-T) cells therapy targeting CD19, currently available for clinical use and offering durable responses in selected patients, both in first line as well as in relapsed/refractory (R/R) disease. Radiotherapy continues to play a critical role in the management of localized disease or as an adjunct to systemic therapy, particularly in early-stage HL and NHL, or for palliation in advanced cases [6–8].

Despite these recent therapeutic advances, R/R lymphomas remain difficult to treat, underscoring the need of keeping supporting research on the identification of new molecular targets and on the design of personalized treatment strategies.

Lymphomagenesis is driven by a variety of molecular alterations, causing the dysregulation of intracellular signaling pathways and epigenetic modifications. These disruptions contribute to malignant transformation, disease progression, and therapeutic resistance. Recent studies have highlighted several molecular targets for therapeutic intervention, among which the serine/threonine kinases CK1α and CK2 have attracted the attention of lymphoma researchers due to their pivotal roles in oncogenic signaling cascades involved in lymphoma pathogenesis. Indeed, a growing number of studies have recently demonstrated that these kinases could play important roles in lymphoma pathobiology [9–12] .

The perturbation of epigenetic mechanisms, such as DNA methylation, histone modifications, and non-coding RNA regulation, which intersect with many diverse signaling networks, adds further layers of complexity to the pathogenesis of lymphomas offering novel points of therapeutic leverage.

This review will provide an overview of the biological functions of CK1α, CK2 and other kinases, and discuss their potential as therapeutic targets in B-cell lymphoma. We will describe the molecular interplay between cell signaling and epigenetic modifications that are regulated by these kinases, with a particular emphasis on the NF-κB, PI3K/AKT, and Wnt/β-catenin pathways. Lastly, we will explore current and emerging therapeutic strategies aimed at targeting these pathways, including the use of kinase inhibitors and other innovative approaches targeting epigenetics regulators. Relevant studies were identified through major scientific databases and selected according to their relevance to the interplay between signaling pathways and epigenetic regulation in hematologic malignancies. Mechanistic, preclinical, and clinical studies that provide meaningful insights into kinase-driven signaling and epigenetic dynamics were included. Given the heterogeneity of lymphoma subtypes and experimental models, discrepancies among studies were carefully considered and interpreted in the context of biological background. The selected literature was subsequently organized into thematic sections centered on key signaling cascades and epigenetic regulators, with the aim of providing an integrated framework that links pivotal signal transduction pathways to epigenetic plasticity and highlights potential opportunities for combinatorial therapeutic strategies.

## Protein kinases: CK1α and CK2

Protein kinases regulate a broad spectrum of cellular processes by catalyzing the phosphorylation of substrate proteins, a key post-translational modification (PTM) that modulates protein activity, stability, and interactions. It is well established that phosphorylation acts as a molecular switch in cell proliferation, apoptosis, motility and differentiation [13, 14].

Aberrant kinase activity is frequently associated with oncogenesis, as dysregulated phosphorylation can drive unchecked cell division, survival, and metastasis [13].

The human kinome comprises over 500 protein kinases, each with unique substrate specificities and subcellular localizations [14]. Among these, the serine/threonine kinases CK1α and CK2 are of particular interest due to their broad regulatory roles in cellular homeostasis. Though initially characterized by their *in vitro* ability to phosphorylate casein, a function now considered non-physiological, CK1α and CK2 have, since then, been recognized as highly conserved, ubiquitously expressed, and constitutively active kinases with pleiotropic functions [10, 11, 15].

**Fig. 1:**
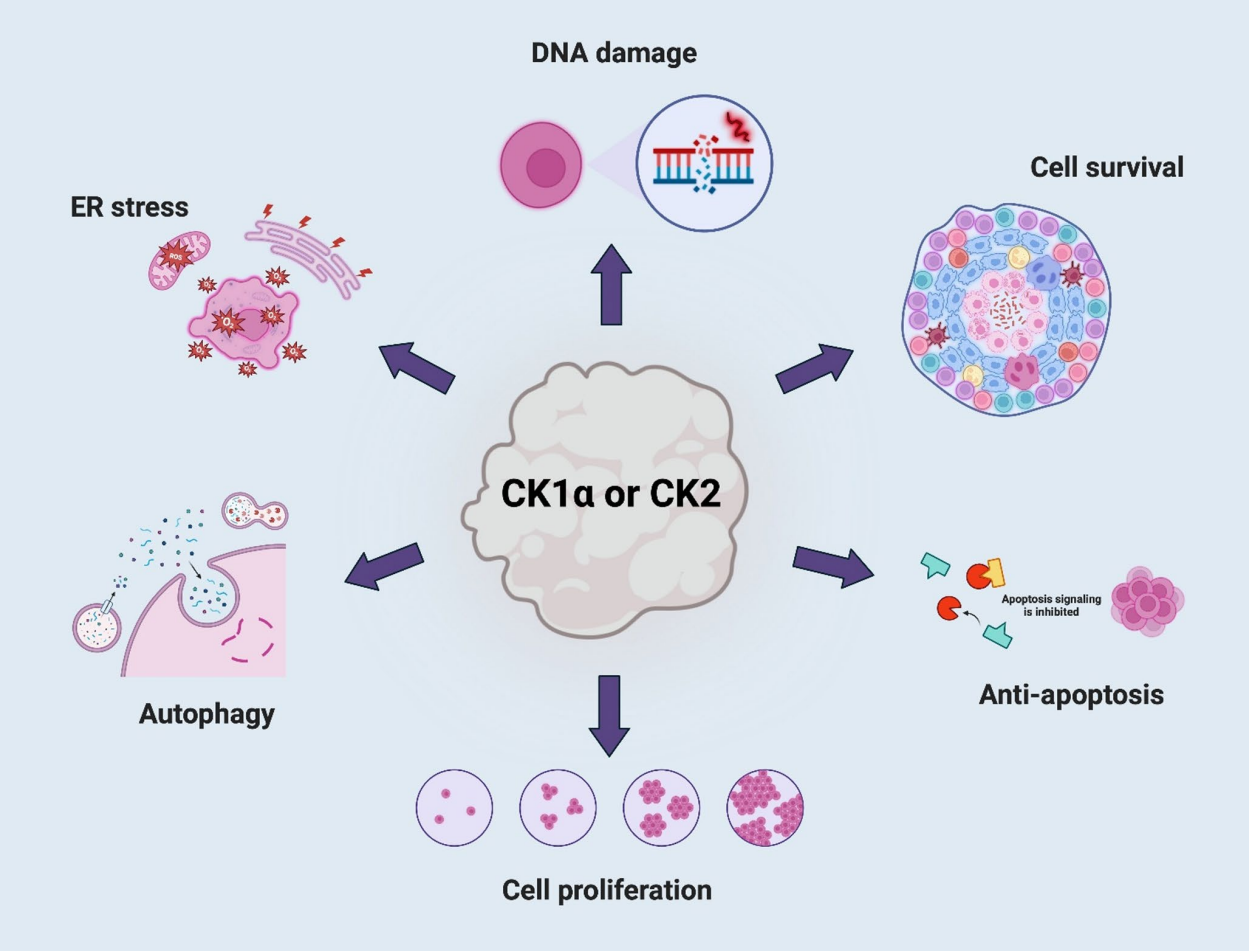
CK1α and CK2 are critical regulators of multiple cellular pathways that support the tumor phenotype. Overview of the cellular processes influenced by CK1α and CK2 in hematologic malignancies. CK1α and CK2 regulate a broad spectrum of cellular pathways, including cell cycle progression, apoptosis, autophagy, DNA damage response, and cellular stress signaling, thereby contributing to the maintenance of cellular homeostasis [9, 10, 18]. Created with BioRender.com

Both kinases modulate key oncogenic pathways involved in cell cycle regulation, DNA repair, and cell survival, and are implicated in the pathophysiology of hematologic malignancies. Notably, CK1α and CK2 also influence microenvironment [16], as well as stress response signaling, including pathways associated with DNA damage, autophagy, and proteotoxic stress (Fig. 1), further linking their activity to tumor maintenance and therapy resistance [11, 17]. Notably, their dysregulation is often linked to various cancers, underscoring their importance as potential therapeutic targets for cancer treatment [10, 14].

### Structural and functional characteristics of CK1α in hematologic malignancies

Protein kinase CK1 alpha (CK1α) is one of seven isoforms of the CK1 family of serine/threonine kinases (α, β, γ1, γ2, γ3, δ, and ε), all of which share a conserved N-terminal kinase domain and exhibit similar substrate preferences [19]. CK1α is encoded by the *CSNK1A1* gene located on chromosome 5q32 and is expressed in four alternatively spliced transcript variants, suggesting functional diversity at the post-transcriptional level.

CK1α functions as a phosphate-directed kinase, typically recognizing substrates that have been pre-phosphorylated at specific positions. Its canonical substrate motif involves a phospho-serine or phospho-threonine residue at the − 3 or − 4 position relative to the targeted serine/threonine site (pS/T-X-pS/T) [20], though non-canonical motifs can also be phosphorylated. This substrate specificity allows CK1α to precisely regulate diverse cellular processes through site-selective phosphorylation. Structurally, CK1α operates as a monomeric enzyme, functioning independently rather than within multi-protein complexes. In hematologic malignancies, CK1α has been found frequently overexpressed, particularly in DLBCL [21], MCL [22], and Multiple Myeloma (MM) [17, 23, 24]. Its aberrant expression influences sensitivity to standard therapies, including lenalidomide, bortezomib, and B-cell receptor (BCR) pathway inhibitors [22, 25, 26], underscoring its potential as both a biomarker and a therapeutic target in blood cancers.

### Structural and Functional Features of CK2 in Hematologic Malignancies

Protein kinase CK2 (CK2) is a highly conserved, constitutively active serine/threonine kinase that plays a critical role in numerous cellular processes, including proliferation, survival, apoptosis, and tumorigenesis [9]. In humans, CK2 functions predominantly as a tetrameric complex comprising two catalytic subunits (CK2α and/or CK2α′, encoded by *CSNK2A1* and *CSNK2A2* genes, respectively) and two regulatory subunits (CK2β, encoded by the *CSNK2B* gene) [27]. The CK2α subunit contains an active site located between its N- and C-terminal domains, while the CK2β subunit enhances kinase activity and determines substrate specificity through its C-terminal interaction with the catalytic subunit [27, 28]. Notably, CK2α can function independently of CK2β [18, 28, 29]. CK2 preferentially phosphorylates serine/threonine residues in substrates that follow a consensus motif characterized by an acidic residue (Asp or Glu) at the + 1 to + 3 position (S/T-X-X-Ac), enabling precise substrate targeting [9, 28]. Both CK2α and CK2β have been implicated in B-cell development [30, 31] and influence erythropoiesis and hematopoietic stem cell maintenance [32, 33].

CK2 has been found frequently overexpressed in B cell–derived malignancies, including MM [34, 35], MCL [35], DLBCL, FL, Burkitt lymphoma (BL) [36], and HL [37], in which it sustains oncogenic signaling pathways critical for tumor cell proliferation and survival.

In MM, CK2 also supports tumor–microenvironment interactions, including osteoclastogenesis, further contributing to disease progression [38].

Importantly, CK2 expression has been linked to therapeutic resistance. In MM and various lymphomas, CK2 modulates cellular responses to bortezomib [35], ER stress-inducing agents [39], BCL2 inhibitor Venetoclax [40], and BCR pathway inhibitors, such as Ibrutinib and Fostamatinib [40, 41]. These findings underscore CK2’s role as a central regulator of both cell-intrinsic and microenvironment-driven oncogenic programs and support its candidacy as a therapeutic target in hematologic malignancies.

### Protein kinases CK1α and CK2: the paradigm of “non-oncogene addition”

Malignant transformation of B lymphocytes often results from genetic alterations affecting oncogenes that disrupt normal cell development and differentiation. However, beyond oncogene activation, B lymphoma cells frequently rely on a broader set of molecular mechanisms for survival, including pathways not directly involved in tumor initiation. This phenomenon, termed non-oncogene addiction, refers to the dependence of cancer cells on genes and pathways that are not inherently oncogenic but are essential for sustaining malignant phenotypes [42, 43]. Cancer cells acquire vulnerabilities through the rewiring of signaling networks that manage key stress responses, such as metabolic dysregulation, proteotoxic and oxidative stress, and DNA damage, hallmarks of cancer that activated due to rapid proliferation and the hostile tumor microenvironment [44, 45]. These stress-adaptive mechanisms frequently involve constitutive activity or overexpression of regulatory proteins that, in normal cells, are either dispensable or tightly controlled. Targeting these processes offers a therapeutic window, as cancer cells are more dependent on them than their healthy counterparts [46, 47].

Among such regulatory proteins, protein kinase CK2 stands out as a central player. CK2 is consistently overexpressed in hematologic malignancies and contributes to pro-survival, anti-apoptotic, pro-inflammatory, and drug resistance pathways, making it a key component of the onco-kinome [46, 48]. Its activity promotes oncogene expression, hampers tumor suppressors and supports hallmarks of cancer such as angiogenesis and multidrug resistance, underscoring its role in tumor maintenance. Despite its oncogenic influence, activating mutations in CK2-encoding genes (*CSNK2A1*, *CSNK2A2*, *CSNK2B*) are rare, suggesting that its dysregulation arises from post-transcriptional or translational mechanisms [9]. Deletions of the *CSNK2B* regulatory subunit with subsequent decrease in gene expression level, occurring in approximately 15% of relapsed DLBCL cases, may result in increased enzymatic activity [49].

In contrast, CK1α, though less directly involved in non-oncogene addiction, has been identified as a “conditionally essential malignant gene” [21]. Its role in cancer is particularly evident in specific hematologic malignancies. Somatic mutations in *CSNK1A1* are found in a subset of myelodysplastic syndromes (MDS) with del(5q), where CK1α haploinsufficiency promotes clonal expansion through the activation of β-catenin and deregulation of TP53 signaling [50]. Furthermore, CK1α overexpression has been observed in DLBCL, MCL, and MM, where it modulates treatment responses [21–24, 51].

Notably, despite the lack of frequent activating mutations, minor alterations in CK1α or CK2 activity can profoundly affect lymphoma pathogenesis and therapeutic response. Thus, these kinases exemplify prototypical regulators of both oncogenic and non-oncogenic pathways that ensure survival, proliferation, and adaptation, making them attractive candidates for targeted therapy in hematologic malignancies.

### Role of CK1α and CK2 in B-cell derived malignancies

Both CK1α and CK2 are involved in regulating key signaling pathways that contribute to the pathogenesis of B-cell derived tumors [9].

In DLBCL, MCL as well as in MM, CK2 has been shown to activate NF-κB signaling, which is a major driver of immune cell survival and proliferation [34, 35, 40]. Dysregulated CK2 activity leads to persistent NF-κB activation, contributing to the survival and expansion of lymphoma cells. Moreover, CK2 interacts with key signaling pathways such as PI3K/AKT [40], JAK/STAT [35] and Wnt/β-catenin [9], further enhancing cell proliferation and resistance to apoptosis.

CK1α plays a pivotal role in regulating the Wnt/β-catenin pathway, a key signaling cascade that impacts on cell fate, cell proliferation and survival. In the context of lymphoma, aberrant activation of this pathway contributes to malignant progression, particularly in T-cell lymphomas and various B-cell malignancies [24]. CK1α regulates this pathway primarily through the phosphorylation of β-catenin, which governs its stability and subcellular localization. Under normal conditions, CK1α participates in the formation of the β-catenin destruction complex by phosphorylating β-catenin on Ser45, which primes it for phosphorylation by GSK3β on Ser33, Ser37, and Thr41 [25, 52]. CK1α has been found overexpressed in MM plasma cells [25, 53] and in MCL B lymphocytes [22] in which it displays a peculiar localization pattern. CK1α has a dual function in the control of β-catenin signaling pathway in MM cells and in stromal cells. While it sustains β-catenin expression in MM plasma cells [25], it lowers its expression in stromal cells, negatively affecting the osteoblastic potential of stromal cells [26]. Therefore, CK1α inactivation could have a dual beneficial effect in MM, restraining plasma cell proliferation and survival on one side, and mitigating MM associated bone disease on the other side. Moreover, CK1α has been implicated also in autophagy regulation in MM [23]. CK1α dependent regulation of AKT signaling in MM [25] and in MCL [22] as well as the regulation of the CBM1 complex and NF-κB in DLBCL [21] and in MCL [22], define CK1α as an important regulator of BCR signaling and consequently inhibitors’ sensitivity in lymphoma.

Table 1 summarizes CK1α or CK2 regulated signaling cascades in B cell derived hematological malignancies

**Table 1:** CK1α and CK2 modulate signaling pathways that sustain proliferation and survival in hematological malignancies

Protein Kinase	Signaling Pathway	Molecular Targets	Hematological Malignancies
CK1α	PI3K/AKT	AKT	MM
CK1α	NF-κB	CARD11, MALT1, IKKα/β, RelA/p65	DLBCL, MCL
CK1α	Wnt/β-catenin	β-catenin	MM
CK2	PI3K/AKT	AKT, PTEN	B-ALL, CLL, LSCs, DLBCL
CK2	NF-κB	RelA/p65	MM, MCL, DLBCL, BL, ALL
CK2	Wnt/β-catenin	β-catenin, Dishevelled (DVL)	CLL

## The importance of epigenetics in the treatment of lymphoma

### Epigenetic dysregulation in cancer: overview

Advances in the understanding of tumorigenesis have revealed that, beyond genetic mutations and deletions, epigenetic dysregulation plays a fundamental role in the initiation and progression of malignancies. While cancer was historically considered a genetic disease, epigenetic alterations, such as histone modifications, DNA methylation, non-coding RNA (ncRNA) activity, and chromatin remodeling, are now increasingly recognized as critical contributors to tumor biology [44, 54].

Epigenetic mechanisms regulate gene expression without altering the underlying DNA sequence. Among these, histone acetylation is one of the most dysregulated processes. It is mediated by histone acetyltransferases (HATs) and histone deacetylases (HDACs), which influence chromatin structure and gene accessibility. Aberrant HDAC activity is frequently associated with suppression of tumor suppressor genes and activation of oncogenes [55, 56].

DNA methylation, particularly the hypermethylation of cytosine-phosphate-guanine (CpG) islands within gene promoter regions, is another epigenetic modification that leads to transcriptional silencing of tumor suppressor genes. This process is catalyzed by DNA methyltransferases (DNMTs) and represents a hallmark of cancer epigenetics [57].

Non-coding RNAs (ncRNAs), including microRNAs (miRNAs), small interfering RNAs (siRNAs), long non-coding RNAs (lncRNAs), PIWI-interacting RNAs (piRNAs), and circular RNAs (circRNAs), regulate gene expression at multiple levels, including transcription, RNA splicing, and translation. They also participate in epigenetic regulation by guiding chromatin remodeling and recruiting transcriptional complexes [58–60].

Chromatin remodeling is a dynamic process that dictates DNA accessibility and gene expression. ATP-dependent chromatin remodeling complexes such as switch/sucrose non-fermentable (SWI/SNF), four major families such as imitation switch (ISWI), chromodomain helicase DNA-binding (CHD), and INO80 Complex ATPase Subunit (INO80) reconfigure nucleosome positioning and are essential for maintaining cellular identity. These complexes work synergistically with histone modifiers and ncRNAs to orchestrate chromatin architecture in response to cellular cues [61].

Given their reversible nature and functional significance, epigenetic alterations represent promising therapeutic targets. Inhibitors of HDACs and DNMTs are already in clinical use or under investigation, underscoring the potential of epigenetic therapy to restore normal gene expression and counteract malignant transformation.

Table 2 describes epigenetic mechanisms and their role in cell biology.

**Table 2:** Epigenetic mechanisms and their biological impact

Epigenetic Mechanism	Key Components	Effect on Gene Expression	Biological Function
DNA Methylation	DNA methyltransferases (DNMT1, DNMT3A/B); 5-methylcytosine	Typically represses gene transcription	Genomic imprinting, X-chromosome inactivation, transposon silencing, cell differentiation
Histone Modification	HATs, HDACs, HMTs, HDMs (e.g., acetylation, methylation, phosphorylation, ubiquitination)	Can activate or repress depending on chemical modification	Regulation of chromatin structure and transcriptional accessibility
Non-coding RNAs (ncRNAs)	miRNAs, siRNAs, lncRNAs, piRNAs, circRNAs	Post-transcriptional gene silencing or scaffolding for epigenetic complexes	Modulation of mRNA stability/translation; recruitment of epigenetic modifiers
Chromatin Reorganization	ATP-dependent chromatin remodeling complexes (e.g., SWI/SNF, ISWI, CHD, INO80)	Alters nucleosome positioning and chromatin compaction	Facilitation or restriction of the transcription machinery access to DNA

In the following subsections, we will provide a detailed overview of some epigenetic mechanisms, examining their roles in the pathogenesis and progression of lymphomas.

### DNA methyltransferases and epigenetic dysregulation in hematologic malignancies

DNA methylation is a pivotal epigenetic modification that governs gene expression by regulating the activity of DNA regulatory elements, particularly CpG islands located in promoter regions [62]. This process is orchestrated by the DNA methyltransferase (DNMT) family, which uses S-adenosyl-methionine (SAM) as a methyl donor.

The core mammalian DNMT family consists of DNMT1, DNMT3A, DNMT3B, and the catalytically inactive regulatory factor DNMT3L; DNMT2 (TRDMT1), although structurally related, primarily functions in RNA methylation and possibly DNA repair [63–65].

Structurally, DNMTs possess a C-terminal catalytic domain, which contains ten conserved motifs responsible for DNA binding, base flipping, and methyl transfer, and a larger N-terminal regulatory domain that mediates nuclear localization, protein–protein interactions, chromatin association, post-translational modifications (PTMs), and interactions with non-coding RNAs [66–68]. DNMT1 is traditionally considered the maintenance methyltransferase, ensuring fidelity of methylation patterns during DNA replication, whereas DNMT3A and DNMT3B function as *de novo* methyltransferases, establishing new methylation marks during development. However, recent evidence suggests functional overlap, with DNMT1 also participating in *de novo* methylation and DNMT3A/3B contributing to maintenance activity [63, 69].

Aberrant DNMT expression has been implicated in B-cell lymphomas and other hematologic malignancies. In MCL, upregulated DNMT1 contributes to the silencing of tumor suppressor genes via hypermethylation, supporting malignant progression [70]. In BL, MYC directly induces DNMT1 and DNMT3B expression, linking oncogenic transcriptional programs to epigenetic silencing [71, 72]. DLBCL exhibits overexpression of DNMT1, DNMT3A, and DNMT3B, which correlates with poor clinical outcomes, likely due to widespread promoter hypermethylation and repression of key tumor suppressor genes [73]. While DNMT3L lacks intrinsic catalytic activity, it facilitates *de novo* methylation by interacting with DNMT3A and DNMT3B, particularly during early development [63]. The roles of DNMT3L and DNMT2 remain understudied in hematologic cancers. Decitabine, a DNMT inhibitor, has shown efficacy in reversing these epigenetic alterations [71].

### Histone methylation and transcriptional regulation in cancer

Histone methylation is a key epigenetic mechanism modulating chromatin structure and gene expression. This process is catalyzed by histone methyltransferases (HMTs), which add one, two, or three methyl groups to specific lysine or arginine residues on histone tails. Arginine methylation can be either symmetric (me2s) or asymmetric (me2a), further diversifying the functional outcomes of these modifications [74].

The biological effect of histone methylation is context-dependent and varies according to the target residue (e.g., H3K4, H3K9, H3K27), the degree of methylation (mono-, di-, or tri-) and in the case of arginine, the methylation symmetry.

These methyl marks function as either transcriptionally permissive or repressive signals, regulating access to DNA and chromatin remodeling. For istance, H3K4me3 (trimethylation of histone H3 lysine 4) and H3K79me2 (dimethylation of lysine 79) are enriched at actively transcribed promoters and gene bodies, thereby functioning as activating modifications. On the other hand, H3K27me3 (trimethylation of lysine 27) and H3K9me3 (trimethylation of lysine 9) are typically found at transcriptionally silent regions and heterochromatin, hence representing repressive modifications. Thus, histone methylation serves as a dynamic and reversible signal, contributing to the epigenetic plasticity observed in cancer, including hematologic malignancies. Disruption of HMT activity or aberrant histone methylation patterns can lead to inappropriate silencing of tumor suppressor genes or activation of oncogenes, promoting malignant transformation and disease progression [74].

#### EZH2 and histone methylation in B-cell lymphomas

Enhancer of Zeste Homolog 2 (EZH2) is a key HMT and the catalytic component of the Polycomb Repressive Complex 2 (PRC2). Its main enzymatic function involves catalyzing the H3K27me3, a histone modification strongly associated with transcriptional repression. EZH2-mediated gene silencing is further reinforced through interactions with DNMTs and HDAC1/2, via its cofactor embryonic ectoderm development (EED) [75, 76].

EZH2 is critically involved in germinal center (GC) B-cell biology, where it regulates immunoglobulin affinity maturation and GC development. Genetic deletion or pharmacological inhibition of EZH2 results in impaired GC formation and function.

In B-cell lymphomas, somatic mutations and overexpression of EZH2 are recurrent and functionally significant. EZH2 is highly expressed in germinal center B cells, and activating mutations are frequently found in FL and germinal center diffuse large B-cell lymphoma (GC-DLBCL) [77–79]. The most common somatic mutations, including EZH2^Y641^ and EZH2^A677^ increase PRC2 catalytic activity, thereby promoting an aberrant accumulation of H3K27me3, and silencing tumor suppressor genes [80, 81]. Notably, the EZH2^Y641^ mutation leads to preferential conversion of H3K27me2 to H3K27me3, enhancing chromatin compaction and transcriptional repression.

Moreover, EZH2 dysregulation is closely linked to MYC overexpression. In BL and double-hit lymphoma (DHL), EZH2 cooperates with MYC to promote aggressive growth and survival [82]. In transgenic mouse models, co-expression of mutant EZH2 and MYC significantly accelerates lymphoma development, highlighting their synergistic oncogenic potential [83, 84].

In cell lines and primary tumor specimens harboring the mutation of EZH2 A677 to a glycine (A677G), researchers observed an atypical pattern of histone methylation: elevated H3K27me3 alongside reduced levels of H3K27me1 and H3K27me2, further confirming the mutation’s role in driving epigenetic reprogramming and tumor progression [85].

Given its central role in repressing gene expression, promoting proliferation, and fostering treatment resistance, EZH2 represents a compelling therapeutic target in aggressive B-cell lymphomas. EZH2 inhibitors are currently being explored in clinical trials, aiming to reverse aberrant epigenetic silencing and restore normal gene function.

### Histone acetyltransferases (HATs) in B-cell lymphomas

Histone acetyltransferases (HATs) are enzymes that catalyze the transfer of acetyl groups from acetyl-CoA to the ε-amino group of lysine residues in histone tails. This modification neutralizes the positive charge of lysines, weakening their interaction with negatively charged DNA and promoting a more open chromatin conformation, which facilitates transcriptional activation [86].

HATs are broadly categorized into Type A and Type B. Type A HATs are nuclear and function primarily in chromatin remodeling and gene regulation. They are subdivided into three major families: CREBBP/EP300, MYST (MOZ, Ybf2/Sas3, Sas2, Tip60) and GNAT (Gcn5-related N-acetyltransferases). These enzymes acetylate both the globular core and the N-terminal tails of histones, regulating gene expression in response to cellular signals [86, 87]. Type B HATs operate in the cytoplasm, acetylating newly synthesized histones H3 and H4 to facilitate proper nucleosome assembly. These modifications are later removed during chromatin maturation, and Type B HATs may also acetylate non-histone proteins, contributing to protein stability and function [88–90].

In B-cell lymphomas, particularly DLBCL and FL, recurrent mutations in CREBBP and EP300, members of the Type A HAT family, are frequently observed: CREBBP mutations occur in ~ 15–30% of DLBCL and ~ 40% of FL cases, while EP300 mutations are found in ~ 5–10% of both DLBCL and FL [91–93]. These mutations typically affect a single allele and often disrupt the acetyltransferase domain, leading to loss of HAT activity. The result is impaired acetylation of both histone and non-histone proteins, contributing to oncogenesis. In particular, inactivation of CREBBP/EP300 leads to reduced acetylation of tumor suppressors like P53, dampening their activity and persistent activation of BCL6, a key transcriptional repressor implicated in germinal center maintenance and lymphoma pathogenesis [88, 89, 94]. The dysregulation of HATs in B-cell lymphomas underlines their dual role in maintaining transcriptional homeostasis and suppressing malignancy, positioning them as potential epigenetic therapeutic targets.

### Histone deacetylases (HDACs) in lymphoma pathogenesis

Histone deacetylases (HDACs) are epigenetic regulators that remove acetyl groups from lysine residues on histone and non-histone proteins, thereby counteracting the activity of histone acetyltransferases (HATs). This restores the positive charge on lysines, enhances DNA-histone interactions, and promotes chromatin condensation and transcriptional repression [95]. HDACs are classified into four major classes based on their homology and cofactor requirements [95]:

- Class I (RPD3-like): HDAC1, 2, 3, and 8, primarily nuclear, involved in transcriptional regulation.
- Class II (Hda1-like): HDAC4-7, 9, and 10, shuttle between nucleus and cytoplasm, involved in differentiation and signal transduction.
- Class III (Sirtuins): SIRT1-7 - NAD⁺-dependent enzymes with diverse roles in metabolism and stress response.
- Class IV: HDAC11, shares features with both Class I and II.

HDACs not only regulate gene transcription but also influence other PTMs, such as SUMOylation, ubiquitination, and methylation, thus broadly impacting cellular signaling networks [96].

In germinal center (GC) lymphomas, particularly DLBCL and FL, mutations in CREBBP impair histone acetylation and indirectly promote increased HDAC3 activity, contributing to transcriptional repression of tumor suppressor genes and GC deregulation [88]. Several HDACs have subtype-specific implications in lymphomagenesis. HDAC6 is frequently downregulated in DLBCL (up to 96% of cases), potentially serving as a favorable prognostic marker in DLBCL, while its loss may promote aggressiveness in PTCL [97]. HDAC9 shows copy number gains in ~ 50% of DLBCL cases; preclinical models suggest its role in enhancing BCL6-mediated transcriptional repression, promoting lymphoma progression, and modulating p53 function [98]. HDAC7 exhibits reduced expression in BL, which may indicate a tumor suppressive role. The silencing of HDAC7 may result from promoter hypermethylation or regulatory non-coding RNAs, such as microRNAs [99].

These findings underscore the central role of HDACs in the epigenetic control of lymphomagenesis and highlight their therapeutic relevance, with HDAC inhibitors currently being explored in both preclinical and clinical settings for the treatment of hematologic malignancies.

A recent preclinical study assessed IOR-160, a dual CK2/HDAC (1, 2, 3, 6) inhibitor, for selectivity and antitumor efficacy in triple-negative breast cancer. IOR-160 exhibited high CK2 specificity, broad HDAC inhibition, and no observable toxicity in mice. In xenograft models, it significantly suppressed tumor growth and burden, inhibited AKT phosphorylation, and increased acetylated α-tubulin, consistent with its dual mechanism. Structural analysis confirmed CK2 binding conservation comparable to CX-4945, supporting the potential of IOR-160 as a therapeutic agent, warranting further preclinical and clinical evaluation [100]. Considering that CK2 is overexpressed in B cell lymphomas and that HDACs are involved in the epigenetic control of lymphomagenesis, this novel dual CK2/HDAC inhibitor could deserve future investigation also in these malignancies.

### BET proteins in epigenetic regulation and lymphomagenesis

The bromodomain and extra-terminal domain (BET) family proteins are epigenetic “readers” that recognize acetylated lysine residues on histones and non-histone proteins, translating chromatin modifications into transcriptional responses. The human BET family includes BRD2, BRD3, BRD4, and the testis-specific BRDT [101]. BET proteins contain two conserved N-terminal bromodomains that selectively bind to acetylated lysines and an extra-terminal domain (ET) that facilitates the recruitment of transcriptional co-activators and chromatin-modifying complexes [102]. Through this binding, BET proteins orchestrate transcriptional programs by bridging acetylation marks with transcriptional machinery. Among BET family members, BRD4 has been extensively studied in the context of cancer, especially B-cell lymphomas. BRD4 is a key regulator of MYC, a well-established oncogene commonly dysregulated in lymphoid malignancies [103]. In GCB-DLBCL, ~ 32% of patients exhibit MYC overexpression, often driven by chromosomal translocations involving the MYC locus [104]. Similarly, in BL, MYC is activated through translocations that fuse MYC to immunoglobulin gene enhancers, resulting in constitutive MYC expression and uncontrolled proliferation [105]. In addition to MYC regulation, BRD4 also modulates cell cycle-related genes, including cyclin D1 and CDK4/6, which are essential for G1-S phase progression [106, 107]. This is particularly relevant in MCL, where the hallmark t(11;14)(q13;q32) translocation leads to overexpression of CCND1 (cyclin D1) under control of the immunoglobulin heavy chain promoter, resulting in CDK4/6 activation and disruption of cell cycle control [108]. Given their central role in transcriptional control of oncogenic pathways, BET proteins, particularly BRD4, represent promising therapeutic targets. BET inhibitors (BETi) are currently being investigated in preclinical and clinical trials for the treatment of aggressive lymphomas [109, 110].

## Interplay between cell signaling and epigenetic regulation in hematologic malignancies

Aberrant cell signaling is a central driver of cancer hallmarks, including uncontrolled proliferation, resistance to apoptosis, and increased invasiveness. These dysregulations often stem from imbalances in kinase and phosphatase activity, driven by somatic mutations, gene amplifications, or epigenetic alterations such as DNA methylation and histone modifications [9, 111]. Such modifications can perturb normal kinase activity or transcription factor accessibility, contributing to the persistent activation of oncogenic pathways critical for tumor maintenance and progression.

Concurrently, epigenetic dysregulation alters transcriptional landscapes by modulating transcription factor binding, gene accessibility, and chromatin conformation. These epigenetic changes can synergize with dysregulated signaling, reinforcing malignant phenotypes by suppressing tumor suppressor genes or enhancing oncogene expression.

Emerging evidence suggests that targeting epigenetic regulators may help re-establish normal gene expression and counteract aberrant signaling cascades, particularly in contexts where tumor suppressor silencing and epigenetically driven pathway activation contribute to malignancy [54].

The following sections will explore the role of the kinases CK1α and CK2 in modulating survival and proliferation pathways in lymphomas and how their actions may intersect with epigenetic regulation to sustain oncogenic signaling.

### CK2 role in epigenetics

Many studies have highlighted the involvement of CK2 in both transcriptional and epigenetic regulation. In yeast, CK2 has been shown to influence gene expression through various mechanisms, including chromatin remodeling, histone modifications, and regulation of histone acetylation and deacetylation processes [112]. Specifically, CK2 phosphorylates histone deacetylases HDAC1 and HDAC2, thereby modulating their activity [113–116]. Among class I HDACs, HDAC1 is phosphorylated by several protein kinases at specific residues that regulate its subcellular localization, enzymatic activity, and protein stability. One of the best-characterized and most studied phosphorylation sites is Ser421, which has been reported to be a target of multiple kinases. When CK2 phosphorylates Ser421 together with Ser423, HDAC1 exhibits increased histone deacetylase activity and accumulates in the nucleus in response to neurotoxic stimuli leading to chromatin condensation and transcriptional repression, which may alter neuronal gene expression under stress conditions [114, 117, 118].

In contrast to the effect of HDAC1 phosphorylation at Ser421, phosphorylation of HDAC2 at Ser394, Ser422, and Ser424 by CK2 suppresses its deacetylase activity and promotes proteasomal degradation. Notably, HDAC2 is phosphorylated by CK2 *in vitro*, and CK2α has been shown to physically interact with HDAC2 [114].

Furthermore, Adenuga and Rahman reported that treatment with the CK2 inhibitor 4,5,6,7-tetrabromobenzotriazole (TBB) disrupts the interaction between HDAC1 and HDAC2 [119].

Additionally, CK2 may impact DNA methylation by targeting DNMT3A through phosphorylation [120]. Interestingly, the *de novo* DNA methyltransferase DNMT3a has been reported to undergo phosphorylation at two critical residues, Ser386 and Ser389, by CK2 [120]. This modification reduces the methylation capability of DNMT3a and redirects its localization to heterochromatin [120].

Notably, CK2 plays a critical role in activating and enhancing the DNA-binding specificity of BRD4 by phosphorylating Ser492 and Ser494 [121]. This interaction promotes the transcriptional activity of c-Myc [121], a finding of particular interest given the documented, though not extensively investigated, oncogenic interplay between CK2 and Myc in murine lymphoma models [122]. Moreover, emerging evidence points to a synergistic therapeutic potential when combining CK2 inhibitors with BET inhibitors in cancer treatment [123], confirmed in MCL by preliminary data from our laboratory [124]. Despite these insights, the role of CK2 in regulating transcription and epigenetics within lymphoid malignancies remains largely unexplored.

More recently, Gong *et al*. demonstrated that CK2 phosphorylates SUZ12 at Ser583 in embryonic stem cells, thereby enhancing PRC2 enzymatic activity [125].

The histone-lysine N-methyltransferase EZH2 is not directly regulated by CK2, but rather by AKT. Specifically, AKT-mediated phosphorylation of Ser21 in EZH2 inhibits its catalytic activity, leading to reduced H3K27 methylation [125].

Histone turnover is particularly dynamic at enhancers and promoters, and the loss of histone chaperones results in histone depletion within transcribed regions. Reassembly of chromatin following RNA polymerase II (RNAP II) passage prevents spurious transcription from cryptic promoters. Numerous chaperones, including SPT2, SPT6, Rtt106, FACT, Vps75, Asf1, and HIRA2, contribute to this process [126]. SPT6, an H3–H4 chaperone, associates with the transcription machinery via the phosphorylated RNAP II C-terminal domain, while SPT2 chaperones H3–H4 tetramers and partially overlaps with SPT6, enabling recycling of H3–H4 tetramers during transcription. This recycling prevents incorporation of newly synthesized, highly acetylated histones that could promote cryptic transcription within coding regions. CK2 interacts with and phosphorylates SPT2, disrupting its interaction with SPT6 and modulating its association with coding regions [126]. CK2 also phosphorylates SPT6, regulating chromatin refolding during transcription elongation. Loss of CK2 activity increases incorporation of newly synthesized H3 in transcribed regions, leading to elevated levels of cryptic sense and antisense transcripts. Mutational analysis of CK2 phosphorylation sites in SPT6 demonstrates their critical role in its function during nucleosome reassembly [126].

It has been recently reported that CK2 phosphorylates and stabilizes PRC2 subunits, promoting H3K27 trimethylation. Prolonged cold induces CK2 accumulation, increasing PRC2 levels and enrichment on FLC chromatin, which spreads post-cold to establish a Polycomb-repressed domain. This cold-CK2–PRC2 pathway converts prolonged cold exposure into epigenetic memory during vernalization. CK2 phosphorylation motifs are conserved across plant and animal H3K27 methyltransferases [127].

While a substantial portion of the mechanistic understanding of CK2 in chromatin regulation derives from studies performed in non-lymphoid systems, including yeast and embryonic stem cells, these findings provide important conceptual frameworks for understanding kinase–epigenetic crosstalk.

Besides the knowledge of the epigenetic control of CK2 in physiological condition, the literature about the role of CK2 in epigenetics of lymphoma is limited and the direct applicability to B cell lymphomas requires careful interpretation. However, given the above mentioned CK2 regulated pathways in lymphoma, a connection with CK2 and epigenetics may be envisioned also in this type of cancer. In particular, emerging evidence from our group further supports a functional interplay between CK2 and key epigenetic regulators in B-cell lymphomas. Our preliminary findings indicate a cooperative role between CK2 activity and BET proteins in sustaining lymphoma cell survival [124] and suggest that combined pharmacological targeting of CK2 and EZH2 results in a synergistic induction of lymphoma cell death [128]. Overall, these data suggest a potential role for CK2 as a connecting element between oncogenic signaling cascades and epigenetic regulatory mechanisms.

### NF-κB

By promoting the transcription of several target genes, the NF-κB transcription factors family coordinates the inflammatory response, cell differentiation, proliferation, and survival. Both canonical and non-canonical NF-κB pathways have been linked to the onset and progression of hematological malignancies, particularly lymphoid leukemias and lymphomas, where a wide range of processes lead to the uncontrolled/constitutive activation of NF-κB [129]. NF-κB mutations are rare, and oncogenic signals are often the cause of unregulated activation. The aberrant activation of this pathway is commonly driven by oncogenic signals that promote the persistent activation of upstream pathways, which, in turn, enhance NF-κB-mediated transcriptional activity [129].

NF-κB is regulated by CK1α in two ways. CK1α initially binds to the activating CBM1 (CARD11/BCL10/MALT1) complex, which initiates T-cell receptor-induced NF-κB activation. Conversely, it negatively modulates the pathway through the phosphorylation of CARD11 on Ser608, leading to the termination of the signaling cascade [21]. In addition, CK1α promotes the survival of NF-κB-addicted activated B-cell like diffuse large B-cell lymphoma **(**ABC-DLBCL) cells by initiating the CBM complex and subsequently activating downstream cascades [130]. CK1α is also involved in the activation of CBM complex and NF-κB to sustain MCL cell growth and proliferation [22].

CK2 affects NF-κB activation through two distinct mechanisms. IkBα is phosphorylated at Ser283, Ser289, and Tyr291 by CK2, leading to its ubiquitin-proteasome-dependent degradation and enhancing the transcription of NF-κB target genes [131]. The direct phosphorylation of the NF-κB family member RelA/p65 on Ser529, which enhances its DNA transactivation activity, is another regulatory mechanism [132]. The overexpression of CK2 has been linked to elevated levels of phosphorylated RelA/p65 on Ser529 in MCL [35, 40], DLBCL [41], MM [35] and HL [37]. As evidenced by the decrease in expression of certain NF-κB target genes, this phosphorylation event appears to be reversed when CK2 is blocked pharmacologically and by RNA interference techniques [35, 40]. Moreover, chemically inhibiting CK2 in FL, BL and DLBCL cell lines led to the downmodulation of phosphorylated RelA, which decreased cell proliferation [36].

NF-κB may also be regulated by HDAC inhibitors (HDACis) [111]. It was found that the HDACi Suberoylanilide hydroxamic acid (SAHA) suppresses tumor cell proliferation, angiogenesis, and inflammation, and is currently in clinical trials. In human myeloid cells, SAHA potentiates apoptosis induced by TNF and chemotherapeutic agents, while inhibiting TNF-induced invasion and osteoclastogenesis, both of which require NF-κB activation [133]. It negatively-regulates NF-κB-regulated genes linked to apoptosis, proliferation, and angiogenesis. SAHA inhibits NF-κB activation by blocking the TNF-induced IκBα kinase cascade, without affecting NF-κB DNA binding. These findings suggest that SAHA’s effects are mediated by modulating NF-κB activity, enhancing apoptosis, and inhibiting invasion and osteoclastogenesis [133]. Another study showed that in MCL, the combined treatment with the HDAC inhibitor vorinostat and either BTK or Syk inhibitors led to the downregulation of NF-κB1/p105 and its activated subunit p50, reduced nuclear DNA-binding activity, and decreased phosphorylation of p65, suggesting inhibition of the NF-κB signaling pathway [134]. Deng *et al* demonstrated that the IKK2 inhibitor LY2409881 strongly synergized with anti-lymphoma drugs. Specifically, LY2409881 and the HDAC inhibitor Romidepsin demonstrated high synergy in T- and B-cell lymphoma cell lines. This synergy likely arises from the antagonistic effect of LY2409881 on Romidepsin-mediated activation of NF-κB [135].

EZH2 has been found to play a non-canonical role in maintaining continuous activation of NF-κB target genes in Estrogen receptor-negative (ER-negative) basal-like breast cancer cells. Rather than depending on its histone methyltransferase activity, this function is mediated through a direct interaction with RelA and RelB, which facilitates the transcription of NF-κB-responsive genes. This results in inflammation, self-renewal, and the development of a tumor-initiating cell (TIC) phenotype in breast cancer [136, 137]. Another study demonstrated that, following EBV infection, NF-κB- p65 binds to the transcription start sites (TSS) of both upregulated and downregulated miRNAs. This binding is associated with H3K27me3 and H3K4me3 [138]. Inhibition of the NF-kB pathway hinders modifications in miRNA expression, it reduces NF-kB binding and prevents the associated histone modifications near the TSS of these miRNAs. These changes in expression also occurred in DLBCL, which are strongly NF-kB dependent [138]. It has been also shown that dimethylaminoparthenolide (DMAPT), a clinical grade water-soluble analog of parthenolide, can reverse cancer-specific epigenetic abnormalities related to NF-κB activity [139]. This study shows that NF-κB inhibition leads to increased levels of histone methyltransferases, indicating a link between NF-κB activity and epigenetic regulation in cancer [139].

### PI3K/AKT

The PI3K/AKT/mammalian target of the rapamycin (mTOR) pathway is one of the main signaling cascades that regulate fundamental intracellular processes, including cell size, growth, metabolism, motility, and proliferation. It is one of the most frequently over-activated/genetically altered cascades in human cancers and it is currently being studied to develop potential clinical grade inhibitors [140]. It has been reported that CK1α regulates the AKT pathway through the phosphorylation of DEP Domain Containing mTOR Interacting Protein (DEPTOR), an inhibitor of mTOR. The mTOR signaling is activated when DEPTOR is phosphorylated in a CK1α-dependent manner on Ser286, Ser287, and Ser291, which causes its proteasomal degradation [51]. As a result, pharmacological CK1 inhibition raises DEPTOR levels and suppresses mTOR signaling [22, 141]. Moreover, it has been demonstrated that CK1α sustains the Ser473 AKT activating phosphorylation in MCL [22] and in MM [25] and CK1α regulates Ser318 and 321 FOXO3a phosphorylation in MM, modulating autophagy [23].

CK2 is involved in the PI3K/AKT pathway through direct phosphorylation of Ser129 on AKT which promotes AKT catalytic activity [142]. Recently, studies in B-NHL and leukemias report that CK2 is also able to influence this pathway regulating the activating phosphorylation of Ser473 on AKT, supporting lymphoma and leukemia cell survival [31, 32, 40, 143, 144].

Growing evidence indicates that reciprocal regulation of key epigenetic mechanisms and the PI3K/AKT axis may contribute to the oncogenic activity of the PI3K signaling pathway in various tumors [145]. Both DNMT inhibitor 5-aza-2-deoxycytidine (5′-Aza) and siRNA blockage of DNMT1 have been shown to greatly reduce the cellular malignant phenotypes of AKT-dominant tumors [146]. Notably, non-histone proteins are occasionally selected as targets by histone modifiers, potentially for both HATs and HDACs. Phosphorylation of class IIa HDACs by growth factor receptor signaling and the mTORC2 pathway ensures the acetylation of its core component, Rictor, thereby promoting sustained downstream signaling, including the activation of c-Myc. This interplay between mTORC2, class IIa HDACs, and Rictor acetylation establishes a self-reinforcing auto-activation loop in glioblastoma cell lines [145, 147, 148]. Also, the whole H3K4 methylation dynamic depends on the PI3K/AKT pathway [147]. In colorectal cancer, insulin activates AKT signaling, which upregulates WDR5 and enhances H3K4me3-dependent expression of zinc finger protein 407 (ZNF407), thereby facilitating epithelial-mesenchymal transition (EMT) [145]. H3K4me3 levels are elevated in breast tumors with activated phosphatidylinositol-4,5-bisphosphate 3-kinase catalytic subunit alpha (PIK3CA), and PI3K/AKT pathway inhibition decreases promoter-associated H3K4me3. The H3K4 demethylase KDM5A is a target of AKT, and its phosphorylation regulates nuclear localization and promoter binding [149]. These findings propose KDM5A localization and genome occupancy as potential pharmacodynamic markers for PI3K/AKT inhibitor response in breast cancer [149].

Protein arginine methyltransferase 5 (PRMT5) is greatly expressed in lymphomas of B cell origin, such as MCL, GCB-DLBCL, and ABC-DLBCL [150]. It plays a critical role in this malignancy through both epigenetic modifications and PST alterations of DNA repair genes, cell cycle controllers,, members of pro-survival cascades, and RNA splicing factors [150]. Antigen-dependent BCR signaling plays a key role in the MCL phenotype, where activation within the lymphoid microenvironment drives the abnormal growth and survival of malignant cells. Inhibition of PRMT5 restores the expression of the truncated form of the protein tyrosine phosphatase receptor type O (PTPROt) [150, 151]. Functionally, PTPROt acts as a tumor suppressor by attenuating proximal BCR signaling, primarily through the dephosphorylation and inactivation of critical kinases such as SYK, LYN, and downstream AKT pathway components, thereby limiting oncogenic signal transduction. This suggests that PRMT5 epigenetically suppresses PTPROt expression in MCL, contributing to tumorigenesis. BCR signaling induces PRMT5 expression in B-cell lymphoma through a PI3K-AKT-MYC positive feedback loop. These observations are particularly significant, as treatment of MCL cells with a selective small-molecule inhibitor of PRMT5, PRT-382, leads to the upregulation of the PI3K inhibitor, Phosphoinositide-3-kinase interacting protein 1 (PIK3IP1), and the negative regulator of AKT activity, Pleckstrin Homology Like Domain Family A Member 3 (PHLDA3), demonstrating that targeting PRMT5 inhibits multiple stages in the BCR-PI3K/AKT signaling pathway [150, 151]. Moreover, PRMT5 promotes MCL cell survival by directly methylating and modulating the activity of key regulatory proteins such as P53, as well as transcription factors such as E2F1, and by epigenetically silencing negative regulators of the BCR pathway (such as PHLDA3, PTPROt, and PIK3IP1). These findings provide a compelling rationale for targeting PRMT5 as a therapeutic strategy in MCL [150, 151].

### Wnt/β-Catenin

Many processes related to cell proliferation and development are influenced by the Wnt/β-catenin signaling pathway. Abnormal cellular alterations caused by any imbalance in its regulation are contributing factors to cancer and the development of several other diseases [152]. Mutations in the constituents of the Wnt/β-catenin cascade have been discovered in various human malignancies. Protein kinase CK1α is a negative regulator of the Wnt/β-catenin signaling pathway.

The Wnt/β-catenin pathway is actively supported by CK2 activity in several ways and it is necessary for Wnt secretion [153]. CK2 stabilizes β-catenin and recruits Wnt regulators to the target genes by phosphorylating disheveled proteins (DVL) [154] and β-catenin [155]. Wnt signaling-driven malignancies are dynamically influenced by epigenetic regulation. To properly control all the cellular functions that occur throughout development and disease, Wnt signaling is a complicated system that is influenced by a variety of cellular and environmental factors. Positive regulators are either hyperactivated or negative regulators are hypoactivated in Wnt-driven malignancies [156].

According to a recent study, EZH2, lysine-specific histone demethylase 1 (LSD1), DNMT1, and HDAC1 form mutual interactions and engage with SMADs and β-catenin in breast cancer cells. Inhibition of these proteins provokes a reduction in their protein levels, except for HDAC1. These alterations activate neuronal genes [157]. In particular, in neural progenitor cells (NPCs), these enzyme (EZH2, LSD1, DNMTs, and HDACs) inhibition similarly drives neuronal differentiation. Notably, EZH2 stabilizes LSD1, HDAC1, DNMT1, β-catenin, and SMAD2/4 through the enrollment of the deubiquitinase USP7 [157]. A reduction in EZH2 levels enhances the ubiquitination and subsequent degradation of associated proteins, leading to reduced binding of LSD1, HDAC1, and DNMT1 to neuronal gene promoters, consequently diminishing Wnt stimulation [157].

**Fig.2:**
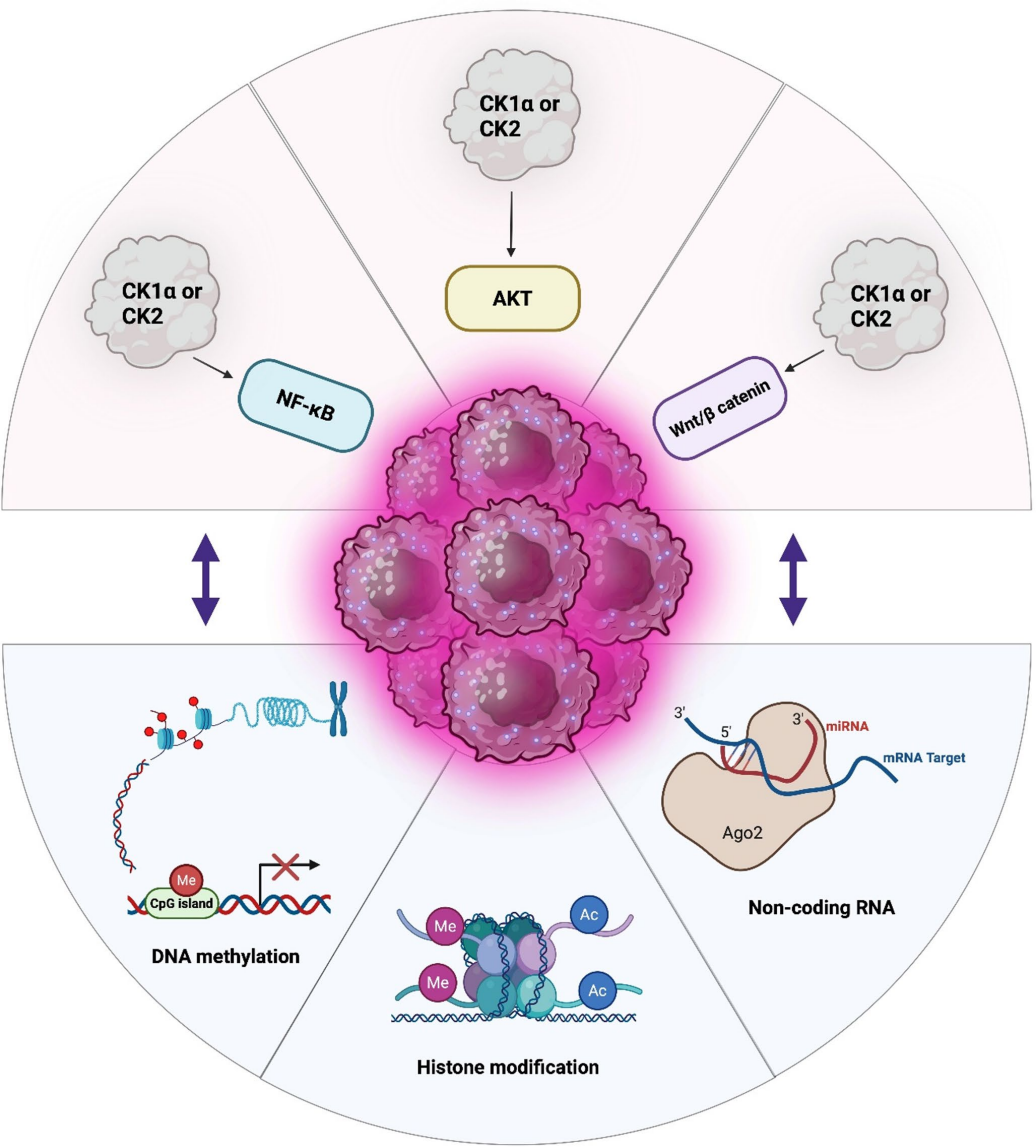
Proposed crosstalk between protein kinases-driven signaling and epigenetic modifications. The figure schematically depicts how, within the neoplastic clone, CK1α and CK2 kinases regulate NF-κB, AKT, and Wnt/β-catenin signaling pathways [9]. The lower part of the scheme illustrates epigenetic regulatory mechanisms, such as DNA methylation, histone modifications, and non-coding RNA-associated modulation. These alterations, including DNA methylation and histone modifications, are also illustrated as potential downstream or cooperating mechanisms of kinase-driven signaling although their integration in B-cell lymphoma is only partially characterized and largely inferred from non-lymphoid models [54, 114, 120]. The bidirectional arrow indicates a proposed crosstalk between signaling pathways and epigenetic regulation contributing to lymphoma progression. Created with BioRender.com

It has been demonstrated that PRMT5 is a key pro-survival factor that stimulates the proliferation of lymphoma cells and is specifically overexpressed in NHL cells. Interestingly, several studies examine the role of PRMT5 in regulating Wnt/β-catenin and AKT/GSK3β signaling in NHL *in vitro* and mouse models [150]. PRMT5 enhances Wnt/β-catenin signaling by directly suppressing the transcription of two critical antagonists, AXIN2 and WIF1, while indirectly activating the AKT/GSK3β signaling pathway. It has been shown that inhibition of PRMT5 through either shRNA-mediated knockdown or treatment with the selective PRMT5 inhibitor CMP-5 results in the re-expression of AXIN2 and WIF1, alongside the inactivation of phospho-AKT (Thr450 and Ser473), and the reactivation of GSK3β [150]. These molecular alterations lead to a reduction in the expression of Wnt/β-catenin target genes such as *CYCLIN D1*, *c-MYC*, and *SURVIVIN*, ultimately inducing lymphoma cell death. Additionally, PRMT5 inhibition results in decreased recruitment of co-activators such as CBP, p300, and MLL1, while simultaneously increasing the recruitment of co-repressors HDAC2 and LSD1 to the promoters of Wnt/β-catenin target genes [150] Figure 2.

## Novel therapeutic approaches in lymphoma:from bench to bedside

Conventional chemotherapy remains the therapeutic backbone of B-cell lymphomas, despite the development of targeted and cellular therapies. In DLBCL, R-CHOP–based chemo-immunotherapy continues to represent the standard first-line treatment, achieving long-term remission in many patients, although outcomes remain suboptimal in biologically high-risk subgroups [158, 159]. Highly aggressive entities such as BL and primary mediastinal B-cell lymphoma require dose-intensive, multi-agent chemotherapy, which remains essential for achieving high cure rates [160, 161]. Nevertheless, despite these advances, there remains a critical need for novel therapeutic targets and strategies to improve outcomes, particularly in relapsed/refractory disease and high-risk patient subgroups.

The abnormal activity of both kinases and phosphatases is critical to cancer development and has become a primary target for therapeutic interventions. Targeted therapies seek to correct this imbalance by modulating kinase and phosphatase functions, aiming at disrupting the abnormal signaling pathways that support cancer cell survival and growth. By restoring proper regulation within these signaling networks, targeted treatments can effectively slow or even arrest the progression of cancer.

A critical kinase in the signaling pathway of B cells is Bruton tyrosine kinase (BTK). It is involved in the B-cell receptor (BCR) signaling cascade, which controls B-cell development, activation, and survival. In B-cell lymphomas, such as MCL, FL and DLBCL, BTK is frequently constitutively activated, leading to proliferation of malignant B cells [162]. The development of covalent BTK inhibitors, such as the first in class Ibrutinib has revolutionized the treatment of these diseases by effectively blocking BTK-mediated signaling and inducing malignant B-cell apoptosis. Ibrutinib, indeed has demonstrated efficacy in B-NHL with the ability to overcome resistance in many cases. Ibrutinib is now used as first line treatment in MCL and Chronic Lymphocytic Leukemia (CLL) [163], however, mechanism of resistance may arise, which could include mutations in BTK, such as kinase “proficient” or kinase “impaired” mutations [164] or in phospholipase Cγ2 (*PLCG2*) [165]. Moreover, activation of alternative downstream BCR signaling, such as PI3K/AKT or NF-kB pathways may be responsible for resistance and therefore treatment failure [166, 167]. From a safety perspective, adverse events such as hemorrhage, atrial fibrillation, hypertension, and infections may occur, leading to treatment discontinuation in a significant proportion of patients [165].

Other second-generation covalent BTK inhibitors, such as Acalabrutinib, Zanubrutinib have been developed to offer improved specificity and reduced side effects, showing promising results. Moreover, the non-covalent inhibitor Pirtobrutinib and others are now approved in relapsed disease after failure of a covalent BTKi [162, 168, 169].

A BCR downstream effector is the activation of PI3K/AKT cascade. Inhibition of the PI3K/AKT pathway has emerged as a promising therapeutic strategy in lymphoma. Among PI3K inhibitors, Copanlisib, Alpelisib, Idelalisib, Duvelisib, and Umbralisib have received approval from the Food and Drug Administration (FDA), however the approval for some of them (Idelalisib, Duvelisib and Umbralisib) have been revoked due to significant safety concerns, primarily a higher risk of death and serious toxicities (such as infections, liver issues, diarrhea/colitis) compared to benefits [170, 171]. Idelalisib, a PI3Kδ inhibitor, has shown significant efficacy in treating FL, small lymphocytic lymphoma (SLL) and indolent NHL. Idelalisib works by blocking PI3Kδ, which is preferentially expressed in B cells, thus inhibiting BCR signaling and promoting apoptosis in lymphoma cells [172]. Duvelisib is an orally available, small molecule inhibitor of the delta and gamma isoforms of phosphatidylinositol 3-kinase (PI3K δ/λ). It is approved for use in SLL and CLL. Duvelisib caused important side effects [173]. Copanlisib is an pan-class I PI3K inhibitor that exhibits strong activity against all four isoforms [174]. It was approved by the FDA 2017 for relapsed FL patients who have experienced at least two previous systemic therapies [170].

Inhibitors of CK1α and CK2 hold considerable promise in the treatment of lymphoma even if they did not reach clinical approval yet. Preclinical studies have demonstrated the effectiveness of small-molecule inhibitors of CK1α and CK2 in reducing lymphoma cell proliferation and inducing cell death. Because of the high degree of similarity among the CK1 family isoforms, it is difficult to create selective inhibitors of CK1α [19]. Nonetheless, recently the compound MU1742 has been identified to effectively inhibit CK1α, and it is the first highly selective and potent inhibitor discovered for this kinase [175]. Moreover, through screening a library of cereblon (CRBN) ligands, SJ7095 was identified as a strong degrader of CK1α, IKZF1, and IKZF3 proteins. Structural optimization led to the development of SJ3149, a selective and effective degrader of CK1α, exhibiting notable activity both *in vitro* and *in vivo.* SJ3149 works by forming a ternary complex with CRBN and DNA damage binding protein 1 (DDB1) to degrade CK1α, confirmed through crystallography and proteomics. It exhibited broad antiproliferative effects across more than 100 cancer cell lines, with activity correlating to the MDM2 inhibitor Nutlin-3a. *In vivo* studies in NSG mice engrafted with MOLM-13 leukemic cells showed effective CK1α degradation and improved pharmacokinetics with intraperitoneal administration [176]. Another CK1α inhibitor, A51, an orally active small molecular inhibitor co-targeting CK1α and the transcriptional kinases CDK7/9, was shown to have the ability to kill Acute Myeloid Leukemia (AML) cells in preclinical mice models [177] and more recently the orally compound BTX-A51 entered in Phase I first clinical trial in advanced MDS and AML [178]. INNO-220, a CRBN-dependent CK1α degrader, was developed using cytokine expression profiling in T cells. It selectively degrades CK1α without affecting IKZF1/3. Lymphoma cells with wild-type p53 and constitutive NF-κB signaling are particularly sensitive to CK1α degradation yet resistant to BTK inhibitors. INNO-220 suppresses NF-κB signaling and activates the p53 pathway, resulting in complete tumor growth inhibition *in vivo*. Mechanistically, it disrupts the CARD11/BCL10/MALT1 complex, inhibiting NF-κB in T cells and CARD11-mutant lymphoma cells. Activation of wild-type p53 alone is sufficient to induce potent cancer cell death even without constitutive NF-κB activity [179].

Several compounds have been identified in relation to CK2, with the majority of these being tiny ligands that inhibit kinase activity at the ATP-binding site. CX-4945 (Silmitasertib) has been shown to be the most appropriate and effective chemical compound, and it reached clinical trial approval for r/r MM (clinical trial#NCT01199718). Research conducted both* in vitro* and *in vivo* has demonstrated that CK2 inhibition with CX-4945 inhibits cell proliferation and triggers apoptosis. In preclinical studies, CX-4945 improved the effectiveness of various drugs in B-NHL. CK2 blockade with CX-4945 increased Bortezomib [35], Venetoclax and Ibrutinib [40] cytotoxicity in MCL, or it worked in concert with Fostamatinib and Ibrutinib, to cause DLBCL cell death [41]. The multifunctional role of CK2 and its effectiveness in preclinical studies support the use of CX-4945 in combination with other therapeutic drugs in the future. Only recently, another selective CK2 kinase inhibitor is the molecule named SGC-CK2-1 (pyrazolo[1,5-a] pyrimidine) [180]. Although SGC-CK2-1 has shown promising preclinical efficacy, clinical data specifically for lymphoma are not present. However, the evidence from cancer research suggests that targeting CK2 may provide therapeutic benefit across various malignancies. Early-phase clinical trials involving CK2 inhibitors that could explore the combination of SGC-CK2-1 with chemotherapy, immunotherapy, or targeted agents are expected to begin soon, as proposed in [180, 181].

Despite these encouraging preclinical findings, the clinical development of CK1α and CK2 inhibitors has been limited by several challenges. These include dose-limiting toxicities due to the broad physiological roles of these kinases, potential off-target effects in normal tissues, and the emergence of adaptive resistance mechanisms that may attenuate therapeutic efficacy over time. Regarding CK1α, poor clinical data are available, and significant challenges are related to isoform specificity within the CK1 family. CK1 inhibitor development remains at an early stage, with limited clinical evidence compared with other kinase targets, and only recently CK1α-targeting compounds entered early-phase clinical evaluation [178], even if BTX-A51 is not CK1α-selective, but is a multi-kinase inhibitor (CK1α, CDK7/9 inhibitor).

CK2 inhibitors show therapeutic promise in oncology and are in more advanced stages in clinical trials. However, CK2 inhibitors clinical translation is limited by the kinase’s ubiquitous expression and pleiotropic functions, insufficient inhibitor selectivity, compensatory signaling and resistance mechanisms. The CK2 inhibitor Silmitasertib has already reached phase I/II. The major concerns are the potential off-target effects and lack of selectivity. Moreover, long-term safety and efficacy are not fully established, and dose optimization represents a key challenge, pointing to the need of a careful titration.

Despite these limitations, both CK1α and CK2 remain attractive therapeutic targets due to their central role in integrating survival signaling and transcriptional regulation in lymphoma cells, and ongoing efforts aim to improve selectivity, combination strategies, and clinical efficacy. The integration of kinase-driven signaling with microenvironmental cues represents a critical aspect of B-cell lymphoma biology. In this context, CK2 operates as intracellular regulator of key survival pathways, including NF-κB and PI3K/AKT, which are also activated by external signals such as the CD40/CD40L axis. Engagement of CD40 on malignant B cells by CD40L-expressing T cells promotes sustained activation of these signaling cascades, thereby reinforcing transcriptional programs that support proliferation, survival, and immune evasion [182]. Importantly, CD40 signaling has also been associated with broader transcriptional and epigenetic reprogramming, contributing to the establishment of a tumor-supportive and immunomodulatory niche [183]. This includes modulation of cytokine production, antigen presentation, and indirectly chromatin-associated regulatory programs that collectively reinforce malignant B-cell fitness within the microenvironment [182, 184]. From a therapeutic perspective, targeting the CD40/CD40L axis, either through agonistic CD40 ligands, recombinant CD40L-based approaches [185], or combinatorial strategies, has been explored as a means to enhance anti-tumor immunity while disrupting pro-survival signaling networks [182]. Integrating such approaches with kinase inhibition and epigenetic therapies may provide a rational framework to simultaneously disrupt intracellular oncogenic signaling and extrinsic microenvironmental support.

Reprogramming cells to improve therapeutic effects in lymphoma represents one way in which epigenetic therapy can simultaneously modulate multiple biological functions. In earlier clinical trials, epigenetic monotherapy demonstrated encouraging outcomes, and multiple epigenetic drugs were authorized to use in the treatment of lymphoma. Moreover, a few therapeutic trials have employed epigenetic treatments in conjunction with immunotherapy and chemotherapy. Among DNMT Inhibitors, Decitabine (DAC) is a deoxyribonucleoside that can be integrated in the DNA to cause hypomethylation of DNA [186, 187]. High amounts of DAC cause cytotoxicity, but modest dosages can reduce toxicity and possibly enhance the targeted impact of DNA hypomethylation by re-expressing tumor suppressor genes during tumor therapy [188]. The effectiveness of combining DAC with a Cisplatin, Cytarabine, and Dexamethasone (DHAP) in treating relapsed/refractory DLBCL (r/r DLBCL) was examined in a phase 4 clinical trial (clinical trial#NCT03579082) but the results are not yet available [54, 188]. DAC and the HDAC inhibitor Cidabendiamide are presently being studied in numerous clinical trials to determine their treatment efficacy in HL (clinical trial#NCT04514081, NCT04233294) [54].

Additionally, the use of new CAR-T therapies in conjunction with the conventional epigenetic drug DAC has increased dramatically. One example of this combination is the treatment of relapsed/refractory B-cell NHL with Decitabine-primed tandem CD19/CD20 CAR-T cells (clinical trial#NCT04697940). Furthermore, sequential administration of low-dose Decitabine with PD-1/CD28-engineered CD19 CAR-T cells is being investigated in relapsed/refractory B-cell lymphoma (r/r B-cell lymphoma) (trial#NCT04850560). An analog of cytidine, Azacitidine can covalently bind to DNMT to prevent DNA methylation and substitute nucleosides in DNA and RNA.

Tazemetostat, the first-in-class EZH2 inhibitor has demonstrated clinical efficacy in EZH2-mutant FL. The compound selectively reduces H3K27 methylation (H3K27me1/2/3), leading to derepression of PRC2 target genes, without affecting total H3 levels or other histone methylation marks. It also induces a modest increase in H3K27 acetylation, an activating epigenetic modification [189]. Inhibiting EZH2 can reverse aberrant histone methylation, leading to the reactivation of tumor suppressor genes and suppression of lymphoma growth [189] overcoming resistance to conventional therapies. It has received FDA approval for EZH2-mutant FL, and further studies are underway to explore its use in combination with other therapies [189]. GSK2816126 is a selective, SAM-competitive inhibitor of EZH2 that reduces global H3K27me3 and reactivates PRC2 target genes. In proliferation assays, B-cell lymphoma lines with EZH2-activating mutations, particularly DLBCL, demonstrated high sensitivity [190]. In a phase I dose-escalation trial of Tazemetostat, the overall response rate (ORR) among B-cell lymphoma patients was 38%, including a partial response in one GCB-DLBCL case lasting 91 days, while six additional patients (five DLBCL and one FL) achieved stable disease [191]. Kagiyama *et al*. demonstrated that OR-S1, a dual EZH1/2 inhibitor and analog of Valemetostat (DS-3201), suppressed Ibrutinib-resistant MCL growth in PDX models by upregulating Cyclin Dependent Kinase Inhibitor 1 C (CDKN1C, also known as p57, KIP2), leading to proliferation arrest, cell cycle blockade, and B-cell differentiation. Valemetostat is currently under evaluation in phase II trials as monotherapy for B-cell lymphomas (trial#NCT04842877) [192].

Based on their molecular composition, HDAC inhibitors fall into four groups: cyclic peptide, benzamide, hydroxamate, and short-chain fatty acid (carboxylate). Although, none of HDAC inhibitors have reached approval only for B cell lymphoma yet, they have been extensively evaluated in B-cell lymphomas, with several agents showing preliminary efficacy. Vorinostat, a pan-HDAC inhibitor, was tested in combination with Cyclophosphamide, Etoposide, Prednisone, and Rituximab in elderly patients with relapsed DLBCL, where it achieved an ORR of 32% (trial#NCT00667615) [174]. Chidamide selectively targets class I HDACs (HDAC1, 2, 3, and 10) by blocking the catalytic pocket, resulting in growth arrest and apoptosis. A large number of clinical trials have been initiated to investigate its therapeutic potential across lymphoma subtypes. In DLBCL specifically, ongoing trials are assessing Chidamide as a single agent (trial#NCT04661943), in combination with R-CHOP compared to placebo (trial#NCT04231448), and in combination regimens incorporating Rituximab, Gemcitabine, and Oxaliplatin (trial#NCT04022005). The isohydroxamic acid-based pan-HDAC inhibitor Abexinostat (CRA-024781), has also demonstrated encouraging activity [193]. Early-phase studies showed a favorable antitumor effect, with a phase II trial reporting an ORR of 56.3% in FL and 21.4% in MCL (trial#NCT00724984). Nevertheless, clinical development of Abexinostat remains limited, and further trials are underway to better define its role, including ongoing evaluations in DLBCL (trial#NCT03936153) and FL (trial#NCT03934567, trial#NCT03600441).

BET proteins function as readers of histone acetylation by binding to acetylated lysine residues in the histone tails. Since oncogene expression super-enhancers frequently exhibit histone acetylation, blocking BET protein binding to chromatin has a major effect on transcription; therefore, the development of several bromodomain inhibitors is ongoing. The exposure of tumor cells to BET inhibitors, such as JQ-1 and INCB 054329, determines a decrease in BRD4 expression and negative regulation of the transcription of downstream targets such as c-MYC, CDK4/6 and RelA [194]. JQ-1 and INCB 054329 may trigger MCL cell lines to undergo apoptosis, proliferation arrest, and overcome their resistance to Ibrutinib [195]. Moreover, treatment with JQ-1 and INCB 054329, caused a reduction in BTK levels [196, 197]. *In vitro* and *in vivo*, JQ-1 and Ibrutinib have demonstrated a synergistic effect in inducing apoptosis [196], indicating that the combination of the BET inhibitor with other anticancer drugs may offer a potential alternative therapeutic approach for MCL patients [196]. In mouse models, it has been demonstrated that the orally bioavailable BET inhibitor RVX2135 promotes the sensitivity of Myc-overexpressing lymphoma cells by reactivating genes silenced by HDACs, thereby highlighting a potential therapeutic strategy based on the synergistic interaction between BET and HDAC inhibition to suppress lymphoma proliferation and induce apoptosis [197].

Anti-CD19 chimeric antigen receptor-modified T-cells (CAR T-cells) have become an innovative and impressive immunotherapy. It has become a promising treatment for relapsed or refractory B-cell lymphomas [198]. Autologous patient-derived genetically engineered T cells expressing a tumor B cell specific surface receptor, such as CD19 are reinfused into the patient, to target and kill lymphoma cells. CAR-T cell therapy has shown remarkable efficacy in treating aggressive B-cell lymphomas, such as DLBCL and FL, particularly in patients who have failed conventional therapies. Clinical studies have shown that the FDA-approved Axicabtagene Ciloleucel (YESCARTA^®^) and Tisagenlecleucel (KYMRIAH^®^) have the potential to trigger humoral responses [199]. In 2020, Tecartus (Brexucabtagene Autoleucel), the third generation CAR-T cell therapy (CD19/FMC63), gained approval for R/R MCL patients’ treatment [200]. Breyanzi (Lisocabtagene Maralecel) (CD19/FMC63) was authorized by the FDA on February 5, 2021, and is recommended for adult patients with R/R large B-cell lymphoma (LBCL) following two or more lines of systemic therapy, comprising DLBCL (including transformed follicular lymphoma), high-grade B-cell lymphoma (HGBCL), primary mediastinal B-cell lymphoma (PMBCL), and 3B grade FL [200].

The efficacy of CAR-T cells is intricately linked to cell signaling pathways, as the activation of these engineered T cells relies on a series of complex intracellular signaling events initiated by antigen recognition. These pathways regulate critical processes such as T cell activation, proliferation, cytokine secretion, and cytotoxic responses. Additionally, epigenetic mechanisms are pivotal in determining the long-term efficacy and persistence of CAR-T cells. Alterations in chromatin structure, DNA methylation, and histone modifications can affect CAR-T cell differentiation, memory formation, and their ability to resist tumor-induced immunosuppression [198]. It has been previously demonstrated that sequencing DNA associated with transcriptionally active (H3K4me2) and repressive (H3K27me3) histone methylation tags reveals significant differences in CAR-T cells from healthy donors (HD) and patients [201].

Metabolic and epigenetic strategies are being utilized to maintain the functionality of minimally differentiated CAR-T cells, which are crucial for long-term efficacy in cancer treatment. One example is the use of the BET protein inhibitor JQ-1, promoting the enrichment of human CD19 CAR-T cells with stem cell memory T (T_SCM_) cells and central memory T (T_CM_) cells. These T-cell subsets are associated with improved long-term persistence and stronger anti-tumor responses [202]. The enhanced *in vivo* persistence and anti-cancer activity of CAR-T cells treated with JQ-1 suggest that manipulating epigenetic and metabolic pathways can improve the effectiveness of CAR-T cell therapies [202, 203].

Another study demonstrated that EZH2 inhibition enhances CAR-T cell efficacy by reprogramming lymphoma models to re-express T cell engagement genes, increasing tumor immunogenicity. EZH2 inhibitors do not impair tumor-reactive or CAR-T cells, while they decrease regulatory T cells, support memory-like CD8⁺ CAR-T phenotypes, and mitigate T cell exhaustion, leading to reduced tumor burden. Intravital imaging confirms enhanced CAR-T cell infiltration and tumor interaction. These findings were validated using syngeneic models mimicking the genetic, epigenetic, and immunological features of human FL and DLBCL [204]. EZH2 inhibition with Tazemetostat enhances anti-CD19 CAR-T cell efficacy in B cell lymphoma by promoting T cell activation, expansion, and tumor infiltration. This is associated with upregulation of genes linked to adhesion, B cell activation, and inflammation. Furthermore, Tazemetostat also improves CAR- and TCR-T cell responses across various hematologic malignancies (MM, AML) and solid tumors (ovarian, prostate cancer and sarcoma) while dual EZH1/EZH2 inhibition with Valemetostat further boosts CAR-T cell function and persistence [205].

In summary, the convergence of CAR-T cell therapy, cell signaling pathways, and epigenetic regulation may present a promising avenue for advancing lymphoma treatment. CAR-T cell efficacy is profoundly influenced by intracellular signaling cascades such as PI3K/AKT and JAK/STAT, which regulate T cell activation, persistence, and exhaustion. Meanwhile, epigenetic mechanisms in tumor and immune cells influence antigen expression, immune evasion, and T cell activity. This combination strategy could overcome tumor immune evasion, boost CAR-T cell efficacy and improve therapeutic responses, leading to more sustained and effective treatments for tumors, including lymphomas.

## Conclusion

Advances in the understanding of lymphoma biology, improved diagnostic techniques, and the development of targeted therapies have greatly enhanced patient outcomes. However, challenges remain in treating aggressive and refractory forms of lymphomas, underscoring the need for continuous research into novel therapeutic targets and personalized treatment strategies. The identification of novel targets such as the protein kinases CK1 and CK2 and epigenetic regulators offers promising avenues for developing more effective therapies. Their roles in critical signaling pathways and epigenetics underscore their potential as therapeutic targets.

Therefore, the identification of targetable protein kinases that are capable of connecting signaling pathways and epigenetic modifications may have a considerable impact in the design of novel therapeutic approaches. Indeed, it is anticipated that a combination of pathway-targeted and epigenetic-targeted therapy might potentiate the anti-lymphoma cytotoxic effects. Future studies should focus on elucidating the precise mechanisms by which these and other kinases contribute to lymphoma pathogenesis while exploring combination therapies that integrate kinase inhibitors with epigenetic modulators.

## Data Availability

No datasets were generated or analysed during the current study.

## Abbreviations

HL: Hodgkin lymphoma
NHL: Non-Hodgkin lymphoma
DLBCL: Diffuse Large B Cell Lymphoma
FL: Follicular Lymphoma
MZL: Marginal Zone Lymphoma
MCL: Mantle Cell Lymphoma
PTCL, NOS: Peripheral T-cell lymphomas, not otherwise specified
ALCL: Anaplastic Large Cell Lymphoma
CK1α: Protein kinase 1 alpha
MM: Multiple Myeloma
BCR: B-cell receptor
BL: Burkitt lymphoma
CK2: Protein kinase 2
ncRNA: Non-coding RNA
HATs: Histone acetyltransferases
HDACs: Histone deacetylases
CpG: Cytosine-phosphate-guanine
DNMTs: DNA methyltransferases
miRNAs: MicroRNAs
siRNAs: Interfering RNAs
lncRNAs: Long non-coding RNAs
piRNAs: PIWI-interacting RNAs
circRNAs: Circular RNAs
SWI/SNF: Switch/sucrose non-fermentable
ISWI: Imitation switch
CHD: Chromodomain helicase DNA-binding
INO80: INO80 Complex ATPase Subunit
SAM: S-adenosyl-methionine
PTMs: Post-translational modifications
HMTs: Histone methyltransferases
H3K4me3: Trimethylation of histone H3 lysine 4
H3K79me2: Dimethylation of lysine 79
H3K27me3: Trimethylation of lysine 27
H3K9me3: Trimethylation of lysine 9
EZH2: Enhancer of Zeste Homolog 2
PRC2: Polycomb Repressive Complex 2
EED: Cofactor embryonic ectoderm development
GC: Germinal center
GC-DLBCL: Germinal center diffuse large B-cell lymphoma
DHL: Double-hit lymphoma
GNAT: Gcn5-related N-acetyltransferases
BET: Bromodomain and extra-terminal domain
CCND1: Cyclin D1
BETi: BET inhibitors
TBB: 4,5,6,7-tetrabromobenzotriazole
RNAP II: RNA polymerase II
HDACis: HDAC inhibitors: TIC: tumor-initiating cell
TSS: Transcription start sites
DMAPT: Dimethylaminoparthenolide
mTOR: Mammalian target of the rapamycin
DEPTOR: Domain Containing mTOR Interacting Protein
ZNF407: Zinc finger protein 407
EMT: Epithelial-mesenchymal transition
PIK3CA: Phosphatidylinositol-4,5-bisphosphate 3-kinase catalytic subunit alpha
PRMT5: Protein arginine methyltransferase 5
ABC-DLBCL: Activated B-cell like diffuse large B-cell lymphoma
PTPROt: Protein tyrosine phosphatase receptor type O
PIK3IP1: Phosphoinositide-3-kinase interacting protein 1
PHLDA3: Pleckstrin Homology Like Domain Family A Member 3
DVL: Disheveled proteins
LSD1: Lysine-specific histone demethylase 1
NPCs: Neural progenitor cells
BTK: Bruton tyrosine
SLL: Small lymphocytic lymphoma
PI3K δ/λ: Delta and gamma isoforms of phosphatidylinositol 3-kinase
CLL: Chronic Lymphocytic Leukemia
CRBN: Cereblon
DDB1: DNA damage binding protein 1
DAC: Decitabine
CX-4945: Silmitasertib
SGC-CK2-1: Pyrazolo[1,5-a] pyrimidine
DHAP: Dexamethasone
AML: Acute Myeloid Leukemia
CDKN1C: Cyclin Dependent Kinase Inhibitor 1 C
ORR: Overall response rate
CRA-024781: Abexinostat
CAR T-cells: Anti-CD19 chimeric antigen receptor-modified T-cells
HD: Healthy donors
LBCL: Large B-cell lymphoma
HGBCL: High-grade B-cell lymphoma
PMBCL: Primary mediastinal B-cell lymphoma
